# Application of Kirchhoff’s Laws to pharmacologic and pharmacokinetic analyses

**DOI:** 10.1016/j.pharmr.2025.100050

**Published:** 2025-03-21

**Authors:** Leslie Z. Benet, Jasleen K. Sodhi, Markus Ville Tiitto, Yue Xiang

**Affiliations:** Department of Bioengineering and Therapeutic Sciences, Schools of Pharmacy and Medicine, University of California San Francisco, San Francisco, California

## Abstract

Recently, we introduced a straightforward approach to derive clearance and rate constant equations, without relying on differential equations, utilizing Kirchhoff’s Laws, a well known physics methodology used to describe rate-defining processes either in series or parallel. Manuscripts from our laboratory have re-examined published experimental data, demonstrating that the Kirchhoff’s Laws methodology can explain data previously considered anomalous, such as the following: (1) all experimental perfused liver clearance data conforming to the equation once thought to represent the unphysiological well stirred model, (2) instances where linear pharmacokinetic systemic bioavailability determinations exceed unity, (3) renal clearance being influenced by drug input processes, (4) statistically significant differences in bioavailability measures between urinary excretion and systemic concentration measurements, and (5) how the long-accepted steady-state clearance approach used in pharmacokinetics for the past half-century leads to unrealistic conclusions about the relationship between liver-to-blood *Kp*_*uu*_ and hepatic availability *F*_*H*_. These findings demonstrate the potential for errors in pharmacokinetic evaluations that rely on differential equations. The Kirchhoff’s Laws approach is applicable to all pharmacokinetic analyses of quality experimental data, both those that align with present pharmacokinetic theory, and those that do not. Although 3 publications have attempted to rebut our position, they fail to address unexplained experimental data, and we detail here why these analyses are invalid. Our discoveries are ongoing. Additionally, we briefly discuss the application of Kirchoff’s Laws to saturable nonlinear kinetics, explaining increased pharmacodynamic response for extended vs immediate release dosage forms, as well as the advantages of successfully formulating high hepatic extraction drugs.

**Significance Statement:**

The Kirchhoff’s Laws approach to deriving clearance equations for linear systems in parallel or in series, independent of differential equations, successfully describes anomalous published pharmacokinetic data that have previously been unexplained. We review 9 experimental outcomes in humans that are newly explained using the Kirchhoff’s Laws approach, including the extension to deriving nonlinear saturable clearance relationships.

## I Introduction

Approximately 2 years ago we made a major discovery, when we recognized that by adapting Kirchhoff’s Laws from physics, it is possible to derive the equations for hepatic clearance independent of differential equations, making no assumptions concerning mechanistic models of hepatic elimination (Pachter et al, 2022). We maintain that the resulting relationship based on Kirchhoff’s Laws is the only valid relationship to describe in vivo organ processes for small molecules when only systemic concentrations are able to be measured. This discovery explained why all valid experimental liver perfusion data were strictly consistent with what had previously been considered the well stirred model (WSM) of hepatic elimination, despite the unphysiological nature of the model (Sodhi et al, 2020b). We then further applied the adapted Kirchhoff’s Laws to derive clearance equations following extravascular drug administration that involves absorption from the site of administration to the systemic circulation via an entering clearance (eg, oral, subcutaneous [SubQ], and intramuscular drug administration) (Benet and Sodhi, 2023). Based on this recognition, we can now elucidate why linear pharmacokinetic systemic bioavailability for slow-release formulations could lead to measurements greater than unity, why in such situations renal clearance would be statistically significantly smaller than following intravenous bolus dosing, and why for such situations bioavailability determination using measurements of unchanged drug in the urine would also be statistically significantly smaller than the systemic concentration bioavailability measures (Wakuda et al, 2024). All these results have been experimentally observed clinically but have remained unexplained, attributed to poor and unreliable experimental procedures and the inherent variability of human studies.

Our approach raises questions about the validity of several basic pharmacokinetic concepts that have been widely taught and utilized in analyzing pharmacokinetic data for the past 80 years. Therefore, it is not surprising that 3 papers have questioned our Kirchhoff’s Laws approach in favor of conventional pharmacokinetic analyses (Korzekwa and Nagar, 2023; Rowland et al, 2023; Siegel, 2023). Notably, what is lacking in these publications is that these investigators have not addressed any of the published experimental pharmacokinetic data that do not fit the current pharmacokinetic theory. Prior to directly responding to these critiques, we instead critically examined the theoretical relationship that underlies the basis for deriving the mechanistic models of hepatic elimination (Benet and Sodhi, 2024a). We demonstrated that by following current theoretical pharmacokinetic assumptions, the mechanistic models of hepatic elimination (the WSM, the parallel tube model [PTM]), the infinite number of dispersion models (DMs) depending on the dispersion number chosen, and the extended clearance model (ECM), which incorporates basolateral hepatic transporters into the WSM, all lead to nonsensical relationships between Kp_uu_, the ratio at steady-state of unbound drug concentration in the liver to the unbound drug concentration in the systemic circulation, and F_H_, the fractional hepatic bioavailability following oral drug administration. Furthermore, for all of these mechanistic models, Kp_uu_ can mathematically never exceed 1.0, an unbelievable outcome that directly results only from following conventional pharmacokinetic theory. In our subsequent manuscript, we then directly responded to the critiques of the above authors (Benet and Sodhi, 2024b), explaining why none of the critical comments were valid.

We are very appreciative of the invitation to submit this review, but our discoveries and new applications are rapidly progressing. We further recognized that if slow absorption could decrease measured renal clearance because of an increase in area under the concentration-time curve as compared to that observed for a comparable intravenous bolus dose, then we might expect to see greater pharmacodynamic effects from such slow-release formulations. This was the subject of our poster at the 2024 meeting of the American Society for Clinical Pharmacology and Therapeutics (Tiitto et al, 2024) and a to-be-published manuscript, which explains why greater pharmacodynamic effects can be observed for an extended-release (ER) oral dosage form versus a comparable dose immediate release (IR) product. Although this paper has not been published, we here describe the statistically significant greater cholesterol-lowering effects of ER versus IR lovastatin. This topic is also related to our publication in the *Journal of Controlled Release* (Benet et al, 2024) which suggests that if a pharmaceutical sponsor could control the rate of drug clearance by manufacturing a well designed slow-release SubQ, lymphatic, transdermal or intramuscular dosage form, then one could administer a high hepatic (or renal) clearance compound without the need to consider any issues of saturability, pharmacogenomics or even enzymatic processes. Until now, we have only addressed linear pharmacokinetic processes, but our accepted poster at the September International Society for the Study of Xenobiotics and the Japanese Society for the Study of Xenobiotics (ISSX/JSSX) meeting in Hawaii, USA (Benet and Sodhi, 2024d) applies Kirchhoff’s Laws to Michaelis-Menten in vivo saturable kinetics. Again, this full paper is presently under review, but we describe the relevant relationships presented in the published abstract in this manuscript. Thus, here in this review, we apply Kirchhoff’s Laws in deriving clearance and rate constants for elimination of the processes above, discuss how these applications successfully explain previously published experimental data thought to be suspect, introduce the basic concepts for our to-be-published additional applications, as well as outline the errors in the publications challenging our approach. We acknowledge that the present differential equation approach to determining the clearance and total rate constants correctly explains the majority of clinical pharmacokinetic analyses. However, we demonstrate that the Kirchhoff’s Laws approach also correctly explains these data, but with a simpler derivation that does not involve differential equations. Importantly, we also point out that although the differential equation approach correctly explains the majority of quality clinical data, it does not account for all quality clinical data. In contrast, the Kirchhoff’s Laws derivation provides a more comprehensive approach that can explain all quality clinical pharmacokinetics data, and this distinction highlights that the Kirchhoff’s Laws approach reviewed here is advantageous to the differential equation approach.

## II The basic concepts: Pharmacokinetics vs chemistry

Rates of reaction are derived for first-order processes following linear force-flow relations as given in eq. 1 as follows:

(1) J=K·f

where J is an outcome such as a driving flux or current, f is the driving force or impetus causing flux, and K is a coefficient of proportionality that determines the magnitude of the outcome response to the input force. We see this relationship for Fick’s law of diffusion, Ohm’s law for electric current, heat transfer relationships and drag force on falling objects.

When applied to chemical rates of reaction, the following equation is obtained as follows:

(2) Rateofreaction=rateconstant·amount

where the proportionality coefficient, the rate constant, k, has units of time^–1^ and the driving force amount, A, has units of mass. The rate of the reaction can be defined using differential equations that include the rate constant and the amount driving the reaction.

In contrast, for in vivo pharmacology, clinical medicine, and pharmacokinetics, the equivalent of eqs. 1 and 2 is as follows:

(3) Rateofreaction=clearance·concentrationdrivingthereaction

where the proportionality coefficient, clearance, CL, has units of volume per time and the driving force concentration has units of mass per volume. Clearance may be calculated by integrating eq. 3 as the amount of drug eliminated or moved divided by the drug exposure driving that elimination or movement (Benet et al, 2021). For an intravenous bolus dose of the drug, clearance may be calculated using eq. 4 as follows:

(4) $$CL=\frac{Amount\;eliminated}{Exposure\;driving\;that\;elimination}=\frac{{Dose}_{iv\;bolus}}{{AUC}_{0\to \infty }}$$

where ${AUC}_{0\to \infty }$ is the area under the systemic concentration time-curve over the time (the exposure) following the bolus dose; for linear systems, the ratio is dose-independent.

It is important to recognize the reasons why one measures concentrations and determines clearance in pharmacology, pharmacokinetics, and clinical medicine. First, in a patient, systemic concentrations are the only easy and convenient available measures of the drug upon which to make drug dosing decisions. Second, for the very great majority of drugs, systemic exposure can be related to measures of efficacy and/or toxicity and the exposure-response relationship is an important tool to support dose optimization and regulatory decisions on dosing selection. Consequently, measures of drug exposure and exposure-response relationships are the basis for all decisions on dose selection in the initial approval studies, as well as the adjustments of drug dosing in disease states, changes related to pharmacogenomics, physiology, drug-drug interactions, and other extrinsic and intrinsic factors (eg, food, immunogenicity). The value of any measured volume of distribution has no relevance to the clearance determination. Third, in pharmacology, pharmacokinetics, and clinical medicine, the coefficient of proportionality between measured concentrations and rate of reaction (the body’s ability to eliminate the drug) is clearance. Therefore, eq. 3 is the appropriate relationship to utilize when drug concentrations (rather than amounts) are measured; however, the approach to converting eq. 2 to eq. 3 by the field of pharmacokinetics has inadvertently introduced errors for pharmacokinetic relationships other than intravenous dosing, as we shall now detail.

Currently, it is commonly believed in our field that converting the chemistry rate of reaction (eq. 2) to the pharmacokinetic rate of reaction (eq. 3) can be achieved by applying a single volume of distribution term for all pharmacokinetic processes. That is, utilize the volume of distribution of drug in the systemic fluids, which we designate as the volume of distribution in the system (*V*_*sys*_), to multiply the eq. 2 driving rate constant, k, and divide the amount of drug in the systemic fluids, A. This operation has been ubiquitous in the field since the initiation of pharmacokinetics. However, in many cases (eg, outside of intravenous bolus drug dosing), this may not be a valid operation. As we shall detail subsequently, in chemistry all reaction rates occur in a fixed volume; therefore, differential equation derivations can be carried out using either concentration or amount measurements to obtain the rate constant coefficients of proportionality, as only one volume of distribution (the fluid volume in the beaker) is relevant for all processes. However, in pharmacokinetics, different processes can occur in different volumes of distribution. For example, absorption from the gut occurs in the volume of distribution for the drug in the gut, absorption from an intramuscular site occurs from the volume of distribution for that site, while elimination from the systemic circulation via metabolism, biliary and/or renal excretion occurs in the systemic volume of distribution determined after an intravenous bolus dose. When deriving differential equations for the amounts of drug in the systemic circulation following oral dosing, that amount-time relationship can only be converted to a concentration-time relationship by dividing by a single volume term, ignoring the difference between the volume of distribution of drug in the gut and the volume of distribution determined following an intravenous bolus dose.

In Table 1, we present a pharmacokinetics timeline related to the ability to measure drug systemic concentrations by chemical methods and the ability to interpret those measurements. We present this timeline to remind readers that all of the basic principles of pharmacokinetics (principles of drug elimination and absorption) were defined in terms of rate constants and differential equations (using both chemical and other methods such as microbiological, ELISA, radioimmune, and thin layer chromatography assays), before the field recognized that to make pharmacokinetics clinically relevant, it was necessary to define elimination in terms of clearance, rather than rate constants. The rate constant derivations were all based on differential equations, as learned in chemistry.

**Table 1:** The pharmacokinetic timeline

Time	Chemical Bio-Analytical Method	Outcome
Pre 1960s	Spectrophotometry	Not sensitive enough to measure systemic concentrations, but useful after urine evaporation. However, because urine amounts directly reflect the systemic concentrations, the rate constants for the systemic concentration-time curve could be determined.
Early 1960s	GLC	Systemic concentrations for some drugs could be determined but were only used to determine rate constants, because the relevance of clearance not yet recognized.
Post 1973	HPLC, LC-MS, LC-MS/MS, etc.	Systemic concentrations of essentially all drugs can be measured. The clinical relevance of determining clearance is recognized but the only method to determine clearance is to derive the chemistry differential equation for the rate of reaction in terms of amounts and rate constants, then divide by the volume of distribution.

GLC, gas-liquid chromatography; HPLC, high-performance liquid chromatography; LC-MS, liquid chromatography–mass spectrometry; LC-MS/MS, liquid chromatography–tandem mass spectrometry.

## III Kirchhoff’s Laws as applied to deriving coefficients of proportionality: Total clearance and the total rate constant of elimination

Just over 2 years ago, we recognized that Kirchhoff’s Laws from physics could be adapted to simply derive equations for total clearance and the total rate constant for elimination, ie, the coefficients of proportionality in eqs. 2 and 3 (Pachter et al, 2022). Taken from that publication, “In 1857 Gustav Kirchhoff published 2 papers, the first titled in English ‘On the Motion of Electricity in Conductors’ (Kirchhoff, 1857a) and the second titled in English ‘On the Motion of Electricity in Wires’ (Kirchhoff, 1857b). In the first paper he demonstrated that when electrical resistors were in parallel, the total conductance (the inverse of resistance) was equal to the sum of the 2 conductances (Kirchhoff, 1857a), which has been subsequently designated as Kirchhoff’s Current Law. In the second paper, he demonstrated that when 2 resistors are in series, the inverse of the total conductance is equal to the sum of the inverse conductance for each resistor (Kirchhoff, 1857b), which has been subsequently designated as Kirchhoff’s Voltage Law.” Our recognition (Pachter et al, 2022) of the relevance of these laws as applied to the coefficients of proportionality in eq. 2 (for determining rate constants for in vitro and in vivo processes) and in eq. 3 (for determining clearance for in vivo processes), is that when 2 or more rate-defining processes occur in parallel, the total value of the measured coefficient of proportionality equals the sum of those rate-defining processes. When 2 or more rate-defining processes are in series, the inverse of the total measured coefficient of proportionality equals the sum of the inverse of those rate-defining processes, as given in eqs. 5 and 6 for clearance.

(5) $${CL}_{total}={CL}_{rate-defining\;parallel\;process\;1}+{CL}_{rate-defining\;parallel\;process\;2}+\ldots \text{.}$$

(6) $$\frac{1}{{CL}_{total}}=\frac{1}{{CL}_{rate-defining\;in\;series\;process\;1}}+\frac{1}{{CL}_{rate-defining\;in\;series\;process\;2}}+\ldots \text{.}$$

When applied to rate constant coefficients of proportionality:

(7) $$k_{total}=k_{rate-defining\;parallel\;process\;1}+k_{rate-defining\;parallel\;process\;2}+\ldots \text{.}$$

(8) $$\frac{1}{k_{total}}=\frac{1}{k_{rate-defining\;in\;series\;process\;1}}+\frac{1}{k_{rate-defining\;in\;series\;process\;2}}+\ldots \text{.}$$

### A Adapted Kirchhoff’s Laws definitions

#### 1 Rate-defining processes

A rate-defining clearance or a rate-defining rate constant is a parameter describing the elimination process where it is possible under certain conditions that the total clearance or total rate constant may be equal to this value. For example, a rate-defining process for hepatic elimination could be hepatic blood flow. Thus, for a very high hepatic clearance drug, total hepatic clearance would equal hepatic blood flow. For a series of metabolic reactions occurring in a beaker, the elimination rate constant (or the clearance) for the parent drug could be the rate-defining process for all subsequent metabolic steps. A rate-defining process is one that potentially alone could define total clearance or a total rate constant, and which can be measured experimentally when it alone defines clearance or when it defines a rate constant. Understanding this definition is essential in the application of Kirchhoff’s Laws to deriving pharmacokinetic coefficients of proportionality. This is not a definition considered by Kirchhoff as he was exclusively concerned with rate-defining processes. In contrast, when applicable to in vivo drug disposition, many processes are affecting drugs that are not rate-defining processes. The critical aspect of our approach is that only rate-defining processes can be combined to determine the overall rate constant for elimination or clearance. Passive permeability into a peripheral compartment from the systemic circulation (and passive permeability from this peripheral compartment back into the systemic circulation) following an intravenous bolus dose, no matter how slow, cannot be a rate-defining process for elimination, ie, clearance and overall elimination rate will never be equal to these intercompartmental reversible passive permeabilities. When hepatic basolateral transporters affect permeability and the active influx is greater than active efflux, this can be a rate-defining process. But not when hepatic efflux is greater than influx. That is, clearance can never be defined in terms of permeability when a measured hepatic basolateral efflux is greater than influx, resulting in a negative value.

The major rate-defining processes in the liver and kidney will be subsequently detailed, including the following: (1) clearance from the delivery site for nonintravenous bolus dosing; (2) organ blood flow; 3) transporter clearance differences, secretion minus reabsorption in the kidney, hepato-basolateral influx minus efflux in the liver; (4) glomerular filtration in the kidney; and (5) metabolism and biliary excretion in the liver.

#### 2 Parallel and in-series processes

In Kirchhoff’s application of parallel and in-series processes to electrical circuits, parallel processes were positionally parallel as well as having no effect on the value of each process. In-series processes followed one another and the first process affected the value of the next in-series processes. However, positional characteristics are not relevant when applying Kirchhoff’s Laws to in vivo clearance and in vitro and in vivo rate constants of elimination. For example, in the liver, there are 2 processes that characterize elimination: (1) metabolism and (2) biliary excretion. Thus, the hepatic intrinsic clearance (CL_H,int_) is the sum of these 2 parallel processes as given in eq. 9.

(9) $${CL}_{H,\mathrm{int}}={CL}_{\mathrm{int},metabolism}+{CL}_{\mathrm{int},biliary\;excretion\;}$$

In pharmacokinetics, the intrinsic hepatic metabolic clearance value has no effect on the transporter biliary clearance value and vice versa (intrinsic clearance [*CL*_*int*_]). They are considered parallel processes, even though positionally metabolism occurs proximal to biliary excretion. Similarly in the kidney, determining renal clearance (CL_R_) independent of kidney blood flow, there are 2 parallel processes governing elimination: (1) glomerular filtration (the product of fraction unbound in the blood, f_uB_, and the glomerular filtration rate, GFR); and (2) the difference between renal transporter related secretion (CL_R,secretion_) and renal reabsorption (CL_R,reabsorption_), which includes both passive and active reabsorption as given in eq. 10.

(10) $${CL}_{R,measureable}={CL}_{filtration}+\left({CL}_{R,secretion}-{CL}_{R,reabsorption}\right)$$

Again, in pharmacokinetics positional placement for parallel processes is not relevant because filtration occurs proximal to the transport processes.

### B The advantages of the adapted Kirchhoff’s Laws derivations

Today the only methodology available to determine the first-order coefficients of proportionality (clearance in pharmacokinetics and rate constants in chemistry) is via solving differential equations. Application of Kirchhoff’s Laws allows one to simply solve for clearance in pharmacokinetics and the overall rate constant in chemistry independent of differential equations, directly by considering only parallel and in-series rate-defining processes as detailed below.

## IV Deriving renal and hepatic clearance relationships using Kirchhoff’s Laws

There are, in fact, very few rate-defining processes of relevance for hepatic and renal clearance. Therefore, the derivations are quite simple and like all clearance equations can be formulated in terms of the in-series entering clearances and the leaving clearances as given in eq. 11.

(11) $$\frac{1}{{CL}_{total}}=\frac{1}{{CL}_{entering}}+\frac{1}{{CL}_{leaving}}$$

CL_leaving_ is defined as a clearance process leading to irreversible elimination from the site where CL_total_ is measured, whereas CL_entering_ is a clearance process for input into the site where CL_total_ is measured. The 3 potential rate-defining processes for renal clearance following intravenous dosing are renal blood flow (Q_R_, the entering clearance) and the in-parallel leaving clearances, glomerular filtration (renal filtration clearance [CL_filtration_]) plus the difference between active tubular secretion and reabsorption (CL_R,secretion_ – CL_R,reabsorption_). These 2 processes are in parallel because the value of CL_filtration_ does affect the value of (CL_R,secretion_ – CL_R,reabsorption_) and vice versa. The 3 potential rate-defining processes for hepatic clearance following intravenous dosing are hepatic blood flow (Q_H_, the entering clearance), and 2 leaving clearances, intrinsic hepatic clearance (CL_H,int_ as given in eq. 9), and the positive difference between hepatic basolateral influx (CL_H,influx_) and efflux (CL_H,efflux_) clearances.

### A Renal clearance

Equation 12 is derived for the entering and leaving clearances as follows:

(12) $$\frac{1}{{CL}_{R}}=\frac{1}{Q_{R}}+\frac{1}{{CL}_{filtration}+\left({CL}_{R,secretion}-{CL}_{R,reabsorption}\right)}$$

where for an intravenous bolus dose, the entering clearance is renal blood flow and the parallel leaving clearances are filtration and the difference between secretion and reabsorption clearances. As we previously pointed out (Benet and Hoener, 2002), there are few drugs exhibiting renal clearance values where renal blood flow (Q_R_ ≈ 1100–1200 mL/min) changes might measurably change renal clearance. However, there are drugs where renal blood flow must be considered as we demonstrate here for metformin. Consider the early study of cimetidine effects on the renal clearance of metformin (Somogyi et al, 1987) where in 7 healthy volunteers, with a mean creatinine clearance of 124 mL/min and an assumed Q_R_ of 1100 mL/min, CL_R_ on day 5 for a daily 250 mg oral dose of metformin HCl was 527 ± 165 mL/min. Assuming that creatinine clearance for metformin, which is not bound to plasma proteins, is the filtration clearance, then the net tubular clearance can be calculated from eq. 12 as follows:

$$\frac{1}{527}=\frac{1}{1100}+\frac{1}{124+\left({CL}_{secretion}-{CL}_{reabsorption}\right)}$$

and $\left({CL}_{secretion}-{CL}_{reabsorption}\right)$_control_ = 888 mL/min. On day 10 of the 250 mg daily oral dosing of metformin HCl, when the subjects had also received 400 mg oral doses of cimetidine twice daily on days 6 through 10, CL_R_ of metformin significantly (*P* = .008) decreased to 378 ± 122 mL/min. Now,

$$\frac{1}{378}=\frac{1}{1100}+\frac{1}{124+\left({CL}_{secretion}-{CL}_{reabsorption}\right)}$$

and $\left({CL}_{secretion}-{CL}_{reabsorption}\right)$_+cimetidine_ = 452 mL/min. Contrast these values with what would be calculated when renal blood flow is not considered (eq. 10), where $\left({CL}_{secretion}-{CL}_{reabsorption}\right)$ is calculated to decrease from 403 to 264 mL/min. That is, the secretory-reabsorption pathway of metformin is markedly more relevant than previously considered and inhibition of secretory pathways causes a larger relative effect than previously considered. This should necessitate a reassessment of in vitro to in vivo predictions of transporter effects. In a planned publication revisiting renal clearance pharmacokinetic concepts in clinical pharmacology and clinical medicine, we will address the importance of considering renal blood flow in analyzing metformin pharmacokinetics, as well as for a number of other drugs where measured renal clearance is greater than 300 mL/min (eg, amiloride, atropine, bretylium, captopril, cimetidine, enalapril, ethambutol, famciclovir, famotidine, hydrochlorothiazide, methicillin, milrinone, minoxidil, neostigmine, nitrofurantoin, nizatidine, oseltamivir, phenylpropanolamine, pindolol, pyridostigmine, and ranitidine).

However, in the great majority of cases Q_R_ >> ${CL}_{filtration}+({CL}_{R,secretion}-{CL}_{R,reabsorption}$) so that renal clearance can be characterized using eq. 10. Under these conditions, it is instructive to compare the Kirchhoff’s Laws derivation (eq. 10) with the differential equation derivation (eq. 13) of renal clearance (renal clearance derived by differential equations [*CL*_*R,diff eq*_]), noting the 4 clinically unmeasurable components of the differential equation derivation [fraction unbound in the renal tubule (f_uTub_), intrinsic renal secretory transport (CL_R,int,sec_), intrinsic renal transporter reabsorption (CL_R,int,reab_) and intrinsic passive renal reabsorption (P_int,reab_)], which are generally ignored when adjusting drug dosing based on renal clearance changes.

(10) $${CL}_{R,measureable}=f_{uB}\cdot GFR+\left({CL}_{R,secretion}-{CL}_{R,reabsorption}\right)$$

(13) $${CL}_{R,diff\;eq}=f_{uB}\cdot GFR+f_{uB}\cdot {CL}_{R,\mathrm{int},secr}-f_{u\;Tub}\cdot \left(P_{\mathrm{int},reab}+{CL}_{R,\mathrm{int},reab}\right)$$

Now consider what can be measured clinically? Only CL_R_, f_uB_, and GFR. And, then what do we learn from eq. 10? If ${CL}_{R,measureable}=f_{uB}\cdot GFR$, then kidney transporter effects appear not to be clinically relevant and drug dosing decisions can be based on changes in GFR (note that as reported by Benet and Hoener (2002), changes in systemic protein binding will not be clinically relevant except for high hepatic clearance drugs following intravenous dosing, eg, lidocaine.) If ${CL}_{R,measureable}>f_{uB}\cdot GFR$, then renal tubular secretion is greater than reabsorption (eg, acyclovir, $\frac{{CL}_{R}}{{CL}_{filtration}}=3.9$; Laskin, 1984) or if ${CL}_{R,measureable}<f_{uB}\cdot GFR$, then renal tubular reabsorption is greater that secretion (eg, omarigliptin, $\frac{{CL}_{R}}{{CL}_{filtration}}=0.44$; Tsuchiya et al, 2017). For the latter 2 outcomes, drug dosing decisions may not be made based only on measured GFR but should consider transporter-related changes. The differential equation approach (eq. 13), and the assumptions required, do not provide such a clear-cut and easy clinically relevant outcome. Although multiple mechanisms can influence reabsorption (urine flow rate, pKa, changes in urine pH), these effects are reflected in the rate-defining process $({CL}_{R,secretion}-{CL}_{R,reabsorption}$).

### B Hepatic clearance

Equation 14 is derived for the entering and leaving clearances as follows:

(14) $$\frac{1}{{CL}_{H}}=\frac{1}{Q_{H}}+\frac{1}{f_{uB}\cdot {CL}_{H,\mathrm{int}}}+\frac{1}{f_{uB}\cdot \left({CL}_{H,\mathrm{int},influx}-{CL}_{H,\mathrm{int},efflux}\right)}$$

where CL_H_ is the hepatic clearance and CL_H,int_ is defined by the parallel processes in eq. 9 and as in the renal clearance derivation, the hepatocellular transporter intrinsic clearances are given as the difference between influx and efflux.

#### 1 When hepatocellular transport is not a rate-defining process

When hepatocellular transport is not a rate-defining process (ie, deleting the third term on the right-hand side of eq. 14), solving eq. 14 yields the following:

(15) $${CL}_{H}=Q_{H}\cdot \frac{f_{uB}\cdot {CL}_{H,\mathrm{int}}}{Q_{H}+f_{uB}\cdot {CL}_{H,\mathrm{int}}}$$

a very familiar equation that has been considered to be the equation for the WSM for the past 50 years (Rowland et al, 1973; Wilkinson and Shand, 1975). Equation 15 was derived here based on Kirchhoff’s Laws making no assumptions related to mechanistic characteristics of hepatic elimination. Equation 15 is organ model-independent, and we maintain that it is the general relationship between *Q*_*H*_, *f*_*u,B*_, and *CL*_*int*_ when only systemic concentrations can be measured. “When only systemic concentrations can be measured” is the critical condition.

The mechanistic model independence of eq. 15 explains why all published quality experimental data in the isolated perfused rat liver (IPRL) align exclusively with this equation, as we reported (Sodhi et al, 2020b). By quality data, we refer to data that are robust and reliable, particularly in studies where experimental inconsistencies have been revealed upon closer examination. IPRL steady-state studies are the only experimental outcomes that can differentiate the WSM from the PTM and the infinite number of intermediary DMs, which depend on the dispersion coefficient assumed. Our review of published IPRL studies supports the generalizability of eq. 15 over the alternatively suggested hepatic disposition models (Sodhi et al, 2020b), by critically examining IPRL data in 4 studies with high-clearance drugs (lidocaine, meperidine, and propranolol), 4 studies with the high-clearance compounds (galactose and taurocholate), and 5 studies where 2 low clearance drugs (diazepam and diclofenac) were made high clearance by manipulating protein binding (as detailed in Table 1 of “Summary of IPRL studies for high-clearance substances” in Sodhi et al, 2020b). We were particularly struck by the fact that the highest cited paper supporting the PTM (Keiding and Chiarantini, 1978) reported several findings that contradict hepatic physiology, including that increasing hepatic blood flow paradoxically led to decreased hepatic clearance for the high extraction ratio compound galactose in 4 of the 10 studies analyzed. This underscores the importance of using well founded data to support or challenge pharmacokinetic theory.

#### 2 When hepatocellular transport is clinically relevant

Under this condition, all 3 parameters on the right side of eq. 14 are considered. However, frequently the relationship is considered when $Q_{H}\gg f_{uB}\cdot {CL}_{H,\mathrm{int}}\;\text{and}\;Q_{H}\gg f_{uB}\cdot \left({CL}_{H,\mathrm{int},influx}-{CL}_{H,\mathrm{int},efflux}\right)$. Then the solution is

(16) $${CL}_{H}=\frac{f_{u,B}\cdot {CL}_{H,\mathrm{int}}\cdot \left({CL}_{H,\mathrm{int},influx}-{CL}_{H,\mathrm{int},efflux}\right)}{{CL}_{H,\mathrm{int}}+\left({CL}_{H,\mathrm{int},influx}-{CL}_{H,\mathrm{int},efflux}\right)}=\frac{f_{u,B}\cdot {CL}_{H,\mathrm{int}}}{1+\frac{{CL}_{H,\mathrm{int}}}{\left({CL}_{H,\mathrm{int},influx}-{CL}_{H,\mathrm{int},efflux}\right)}}$$

From eq. 16, when CL_H,int_ is much greater than $\left({CL}_{H,\mathrm{int},influx}-{CL}_{H,\mathrm{int},efflux}\right)\text{,}$ hepatic basolateral transport is the rate-limiting step; when $\left({CL}_{H,\mathrm{int},influx}-{CL}_{H,\mathrm{int},efflux}\right)$ is much greater that CL_H,int_, hepatic elimination is the rate-limiting step. However, eq. 16 also describes the CL_H_ relationship when both basolateral transport and hepatic elimination are relevant. All of the clinical clearance relationships for statins and other acids with molecular weights greater than 400 can be described by eq. 16. At present, these data are analyzed in terms of the ECM equation (eq. 17), which was initially proposed by Sirianni and Pang (1997) and further derived by many authors, as we reviewed (Benet et al, 2018a):

(17) $${CL}_{H,ECM}=\frac{{CL}_{H,\mathrm{int},influx}\cdot f_{uB}\cdot {CL}_{H,\mathrm{int}}}{\left({CL}_{H,\mathrm{int}}+{CL}_{H,\mathrm{int},efflux}\right)}$$

We will demonstrate the invalidity of eq. 17 in the next section, IV.B.3, but here we compare how these 2 equations treat rate-limiting steps of hepatic elimination. In eq. 17, for hepatic basolateral transport to be rate limiting, ${CL}_{H,\mathrm{int},efflux}$ is zero or negligible, so that CL_H,int_ in the numerator and denominator cancel. Then ${CL}_{H,ECM}=f_{uB}\cdot {CL}_{H,\mathrm{int},influx}$. But why would efflux transport be zero or negligible for hepatocellular transport to be rate-limiting? From eq. 16, if the positive difference between influx and efflux is much smaller than hepatic intrinsic clearance, then, ${CL}_{H}=f_{uB}\cdot \left({CL}_{H,\mathrm{int},\inf\;lux}-{CL}_{H,\mathrm{int},efflux}\right)\text{.}$ Furthermore, from eq. 17, how is it possible for CL_H,ECM_ to be rate-limited by hepatic intrinsic clearance? In contrast, eq. 16 is consistent with ${CL}_{H}=f_{uB}\cdot {CL}_{H,\mathrm{int}}$ when CL_H,int_ is much smaller than the positive difference between influx and efflux.

Furthermore, we now recognize that just as we detailed the relationship between transporter activity and renal clearance, above, the same holds for published analyses of hepatic clearance and transporter activity. Use of eqs. 16 or 17 ignores the effects of hepatic blood flow. We suggest that the extent of transporter interactions on fluvastatin with a hepatic blood clearance of 1134 mL/min (Tse et al, 1992), atorvastatin with a hepatic blood clearance of approximately 1140 mL/min (Gibson et al, 1997), and repaglinide with a hepatic blood clearance of 877 mL/min (Kudo et al, 2013) should be reanalyzed together with potentially other OATP substrates.

#### 3 There is no clinical relevance to the mechanistic models of hepatic elimination

The initial proposed mass balance derivation for the WSM (Rowland et al, 1973), which the senior author of this review coauthored, assumed the steady-state relationship in eq. 18:

(18) $${CL}_{H}\cdot C_{Blood}=Q_{H}\cdot \left(C_{in}-C_{out}\right)={CL}_{H,\mathrm{int}}\cdot C_{H,u}={CL}_{\mathrm{int}}\cdot C_{out,u}$$

where C_Blood_ is the steady-state concentration of total drug in the blood, C_in_ and C_out_ are the blood concentrations of total drug (unbound plus bound) entering and leaving the liver, respectively, C_H,u_ is the average concentration of unbound drug within the liver, which in the WSM is assumed to equal the unbound drug concentration in the liver exiting blood, C_out,u_, due to infinite mixing of the organ. In 2018, we first began to question the validity of eq. 18 (Benet et al, 2018b) questioning why the elimination of the drug from the systemic circulation is a function of hepatic blood flow, but the elimination from the liver is not a function of hepatic blood flow. If clearance measured in blood is rate limited by hepatic blood flow, should not liver clearance also be rate limited by hepatic blood flow?

In fact, variants of eq. 18, as reviewed by Li and Jusko (2022), form the basis of all clearance equations for the WSM, the PTM first presented by Pang and Rowland (1977) and the DMs first presented by Roberts and Rowland (1986), as given in eq. 18a (*C*_*in,u*_ is the concentration of unbound drug in blood entering the liver):

(18a) $${CL}_{H\;\left(WSM,PTM,DM\right)}\cdot C_{Blood}=Q_{H}\cdot \left(C_{in}-C_{out}\right)={CL}_{\mathrm{int},WSM}\cdot C_{out,u}={CL}_{\mathrm{int},PTM}\cdot \frac{{C_{in,u}-C}_{out,u}}{\ln\frac{C_{in,u}}{C_{out,u}}}={CL}_{\mathrm{int},DM}\cdot C_{avg,u,DM}$$

That is, for the 3 mechanistic models of hepatic elimination used in pharmacokinetic evaluations and in making predictions in physiologic-based pharmacokinetic (PBPK) models, it is assumed that the product of the measured steady-state systemic clearance and the steady-state drug concentration measured in blood (left-hand side of eq. 18a) is equal to the product of the average liver concentration at steady-state for that specific model and the intrinsic hepatic clearance for that model.

**Fig. 1:**
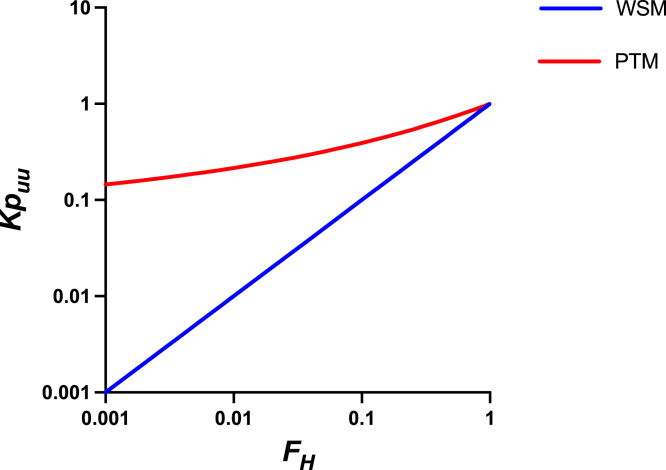
Theoretical relationship between Kp_uu_ and F_H_ for the WSM and PTM If one accepts eq. 18a as valid.

If one chooses to discard the experimental analysis discussed above indicating that no data from quality IPRL studies can be best described by the PTM and DM models (Sodhi et al, 2020b), then it may be considered that there are only theoretical differences between the 2 positions, ie, our position based on Kirchhoff’s Laws asserting that eq. 15 is model-independent and is applicable to all clinical measures of hepatic clearance versus the position of those who support the mechanistic models of hepatic elimination. However, recently we also challenged the theoretical validity of the traditional derivation of mechanistic models of organ elimination (Benet and Sodhi, 2024a). In that publication, we demonstrated that analyzing the mechanistic models of hepatic elimination (and the ECM) in terms of Kp_uu_, the steady-state ratio of unbound concentrations in the liver to unbound systemic concentrations (Di et al, 2021), leads to illogical outcomes, providing strong evidence against the validity of these mechanistic models. We first determined the Kp_uu_ value for each of the mechanistic models and then showed that for each model (WSM, PTM, DM, and ECM) the Kp_uu_ value was mathematically related to F_H_, the model-independent measure of hepatic oral bioavailability ($F_{H}=1-\frac{{CL}_{H}}{Q_{H}})$ with the result that Kp_uu_ values could never exceed unity. Figure 1 depicts the Kp_uu_ vs F_H_ relationship for the WSM and the PTM from Benet and Sodhi (2024a). Although we did not derive the relationship for the infinite number of DMs, lines would always be intermediate to those for the WSM and PTM.

We also showed that for the ECM, Kp_uu_ values were always less than F_H_. We believe that pharmaceutical scientists would not accept these nonsensical relationships between Kp_uu_ and F_H_, that Kp_uu_ can never exceed unity, and that Kp_uu_ is completely independent of any structural and physicochemical characteristics of a drug molecule, as we have recently emphasized (Benet and Sodhi, 2024a,b,c). Accepting the Kirchhoff’s Laws derivation of hepatic clearance (eqs. 15 and 16), eliminates any relationship between Kp_uu_ and F_H_. Therefore, as previously universally accepted, Kp_uu_ is a characteristic specific to each drug molecule, with values that range from below unity to more commonly greater than unity due to the lipophilic nature of most drug molecules (Sodhi et al, 2020a). We believe that this analysis strongly confirms our position that mechanistic models of hepatic elimination provide no benefit in determining the clearance relationship for clinical measures of systemic concentration-time relationships.

We are not suggesting that the mechanistic models of hepatic elimination could not be useful and relevant if one were able to measure intrahepatic concentrations as a function of time at various points in the liver, or even the average concentration within the liver at steady-state, which could be accomplished with animal studies. However, the conclusion as to the relevant model of hepatic elimination based on intrahepatic measurements would provide no useful estimates of hepatic clearance when only systemic concentrations can be measured because then only eq. 14 is valid.

## V The utility of Kirchhoff’s Laws in deriving chemical rate of reaction relationships

**Fig. 2:**
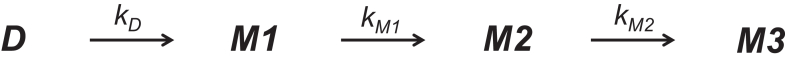
The in-series in vitro linear metabolic steps of a drug in a microsomal mixture.

The unique benefits of the Kirchhoff’s Laws approach are seen in pharmacokinetics, allowing clearance relationships to be derived that cannot be obtained using differential equations due to in vivo processes involving more than one volume of distribution. However, even in chemistry and pharmacokinetic derivations only involving rate constants, the use of Kirchhoff’s Laws provides useful simplifications. Consider the in-series metabolic in vitro first-order reactions adapted from Benet and Sodhi (2023) as depicted in Fig. 2 where 90 *μ*moles of a drug is placed in 20 mL of fluid containing abundant microsomal enzymes and the kinetics of a drug (D) and subsequent in series metabolites, M1, M2, and M3 are measured.

To exemplify the advantage of the Kirchhoff’s Laws approach for linear systems, the question to be addressed for demonstration purposes is what is the mean residence time (MRT) of M2 when the drug is placed in the microsomal mixture? Because this is an in vitro study and metabolites M1 and M2 are available, it is possible to separately measure the rate constant for loss of D (k_D_ = 0.07 h^–1^), loss of M1 (k_M1_ = 0.12 h^–1^, k_M_ is the first-order rate constant for elimination of a metabolite) and loss of M2 (k_M2_ = 0.42 h^–1^). Using the present methodology to determine the MRT of M2, one derives the equation for the concentration-time curve for M2, which requires solving 3 differential equations. Using Laplace transforms and input and disposition functions (Benet, 1972) the following equation would be obtained (time [*t*]):

$$C_{M2}=\frac{k_{D}\cdot k_{M1}\cdot Dose}{\left(k_{M1}-k_{D}\right)\cdot \left(k_{M2}-k_{D}\right)\cdot V}e^{-k_{D}\cdot t}+\frac{k_{D}\cdot k_{M1}\cdot Dose}{\left(k_{D}-k_{M1}\right)\cdot \left(k_{M2}-k_{M1}\right)\cdot V}e^{-k_{M1}\cdot t}+\frac{k_{D}\cdot k_{M1}\cdot Dose}{\left(k_{M1}-k_{M2}\right)\cdot \left(k_{D}-k_{M2}\right)\cdot V}e^{-k_{M2}\cdot t}$$

Substituting the values for the parameters

$$C_{M2}=2.16\;e^{-0.07\cdot t}-2.52{\;e}^{-0.12\cdot t}+0.36\;e^{-0.42\cdot t}$$

One may now calculate the apparent MRT for M2, by determining the area under the moment curve (AUMC) from the sum of the coefficients divided by the exponents squared, and the area under the curve (AUC) from the sum of the coefficients divided by the exponents as reported by Yamaoka et al (1978).

$$\frac{{AUMC}_{M2}}{{AUC}_{M2}}=\frac{\frac{2.16}{0.07^{2}\;}-\frac{2.52}{0.12^{2}}+\frac{0.36}{0.42^{2}}}{\frac{2.16}{0.07}-\frac{2.52}{0.12}+\frac{0.36}{0.42}}=\frac{267.9}{10.71}=25.0\;h$$

That is, even though the measured MRT for M2 when it is placed in the beaker containing the microsomal solution is 2.38 hours (determined as 1/k_M2_), when M2 is measured following drug D being placed in the beaker the MRT of M2 appears to be more than 10-fold greater because the formation of M2 is rate limited by D and M1 disposition.

Alternatively, one may solve for the MRT for M2 using Kirchhoff’s Laws. There are 3 rate-defining processes in series affecting the measured M2 concentrations. Therefore:

$$\frac{1}{M2\;measured\;elimination\;rate\;constant}=\frac{1}{k_{D}}+\frac{1}{k_{M1}}+\frac{1}{k_{M2}}$$

Substituting the values for the parameters

$$\frac{1}{M2\;measured\;elimination\;rate\;constant}=\frac{1}{0.07}+\frac{1}{0.12}+\frac{1}{0.42}=25.0\;h$$

As noted above, the inverse of the M2 measured elimination rate constant is the MRT of metabolite M2 when drug D is placed in the incubation mixture. All of the complicated differential equation derivation steps above are unnecessary using Kirchhoff’s Laws. Thus, although the present differential equation methodology will give the correct amount-time relationships, no matter how many volume terms are relevant with respect to concentration derivations, the Kirchhoff’s Laws approach greatly simplifies the derivation and negates any need for using complex differential equation derivations. Note that the demonstration here exemplifies the advantage of the Kirchhoff’s Laws approach when deriving rate constants both in vitro in chemistry and in pharmacokinetic evaluations and when deriving rate constants in vivo. We review the application to in vivo clearance calculations in the following sections.

## VI Application of Kirchhoff’s Laws to explain previously considered anomalous experimental data following slow drug input processes

Since the beginning of the application of pharmacokinetics to derive differential equations for first-order processes, rates of reaction in terms of measurable systemic drug concentrations were based on chemical kinetic concepts. These equations were then integrated over all time to define the relationship between systemic exposure, available dose, and clearance as given in eq. 4 above, which can be rearranged to

(4) $${AUC}_{0\to \infty ,iv\;bolus}=\frac{Dose}{{CL}_{iv\;bolus}}$$

For an orally administered dose, the numerator is the product of the systemic bioavailability (*F*) and dose.

(19) $${AUC}_{0\to \infty ,oral\;dose}=\frac{F\cdot Dose}{{CL}_{oral\;dose}}$$

For the past century, all teaching of pharmacokinetics and every pharmacokinetic textbook generally assumes that for drugs following linear pharmacokinetics where there is no change in the elimination or distribution rate constants, then ${CL}_{oral\;dose}=$ ${CL}_{iv\;bolus}$. The implication of this assumption is that the input process (the rate of absorption from the gastrointestinal tract) has no effect on the measured AUC and thus *F* may be calculated from the dose-corrected ratio of AUCs of oral/intravenous bolus over all time.

However, as we recently wrote (Benet and Sodhi, 2024b), “It is quite simple to demonstrate that these universally believed assumptions may not always be true. All comprehensive textbooks of pharmacokinetics describe the so-called ‘flip-flop’ model, as we previously reviewed (Garrison et al, 2015), for which the absorption rate constant from the gastrointestinal tract is much slower than the rate constant for elimination from the systemic circulation and therefore, the elimination from the systemic fluids will be rate limited by the slow absorption. Thus, by always assuming that for linear systems ${CL}_{oral\;dose}=$ ${CL}_{iv\;bolus}$, our field for the past 80 years has paradoxically both (a) recognized that the elimination rate from the systemic circulation can be rate-limited by the slow gastrointestinal absorption rate, but assumes that (b) slow drug clearance from the gastrointestinal absorption site does not affect measured systemic rate of elimination.” Our field recognizes that if a drug undergoes sequential metabolism in vivo (metabolic in series steps; eg, drug to metabolite 1 to metabolite 2 to metabolite 3), and the rate constant of elimination of drug to metabolite 1 is very slow, then following dosing of the drug, the measured clearance rate of transformation of metabolite 2 to metabolite 3 will be rate-limited by clearance of the drug (Houston and Taylor, 1984). This is true even when it is known that if we dosed metabolite 2, its measured clearance value would be much higher than the clearance of the drug itself. Thus, we pose the question for an analogous scenario, what is the basis for believing that slow clearance from the gastrointestinal tract would not affect the measured systemic AUC following oral dosing?

Recognizing that for linear systems, measured ${CL}_{oral\;dose}$ may not always be equal to ${CL}_{iv\;bolus}$ due to slow entering clearance from the gastrointestinal tract, this provides the explanation for appreciable published pharmacokinetic results considered previously to be anomalous. In Wakuda et al (2024), we list 32 peer-reviewed studies in humans and animals where systemic bioavailability measures of 1.03 and greater are reported, and often these results were statistically significant. Because there has been no explanation for bioavailability measures being greater than 100% for linear systems until now, in many of these publications, particularly for human studies, the investigators search for explanations that identify the errors or conditions that occurred in their studies (eg, alphabetically: analytical, biliary cycling, binding to apparatus, different studies not giving the same results due to variability, exceedingly high urine flow rates, highly variable concentrations, lack of statistical validation, and saturation of elimination).

Do we believe the explanations, which are usually only investigated and detailed because of the unexplained results? Perhaps yes for some studies, but for some certainly not. In fact, we make no assumption as to the validity of all of the 32 human and animal studies listed in Wakuda et al (2024). Of note, we only listed those studies published in the literature. However, as a consultant to the industry over the years, the senior author of this review has seen the confidential data for many more such studies both in humans and animals, following oral and intravenous studies yielding dose-corrected systemic AUC ratios greater than 1.0 that were never published because no reasonable explanation for the results could be obtained. Here in Table 2, we cite the following: (1) 3 examples where measures of systemic bioavailability are statistically significantly greater than 1 (cimetidine, levetiracetam, ofloxacin and probably additionally treprostinil sodium); (2) 2 human studies following slow oral and SubQ drug administration where renal clearance is markedly lower than renal clearance following intravenous dosing (1-deamino-8-arginine vasopressin and cimetidine), and (3) cilazapril in addition to cimetidine where bioavailability in humans calculated using systemic concentrations is statistically significantly greater than bioavailability calculated using measures of unchanged drug in urine within the same study.

**Table 2:** Measures in humans of systemic bioavailability, urinary measures of bioavailability for the same studies, and measures of renal clearance compared to intravenous dosing exhibiting data not consistent with present pharmacokinetic principles

Drug	Species	Route_X_	AUC_x_/AUC_iv_	CL_R,x_/CL_R,iv_	Reference
1-Deamino-8-arginine vasopressin	Healthy humans	SubQ	1.66^a^	0.76	Fjellestad-Paulsen et al, 1993
Treprostinil sodium	Healthy humans	SubQ	1.13 ± 0.10^b^		Wade et al, 2004
Cimetidine	Healthy humans	Oral	1.11 ± 0.39^c^ U_24,po_/U_24,iv_ = 0.45 ± 0.12^c^ *P* < .001 U_24_ ratio vs AUC_∞_ ratio, paired *t* test	0.63 ± 0.32^c^ *P* < .002 CL_R,po_ vs CL_R,iv_ paired *t* test	Grahnén et al, 1979
Levetiracetam	Healthy humans	Oral	1.09 90% confidence interval 1.05–1.13^d^		Ramael et al, 2006
Ofloxacin	Healthy humans	Oral	1.05 ± 0.07 *P* < .05 AUC_po_ vs AUC_iv_ paired *t* test		Yuk et al, 1991
Cilazapril^e^	Healthy humans	Oral	0.775 ± 0.110 vs U_∞,po_/U_∞±,iv_ = 0.571 ± 0.103 *P* < .001 *t* test^f^		Williams et al, 1989

a The authors explain the high AUC ratio based on the adsorption of the drug to the syringe following intravenous dosing, but this correction still leaves (AUC_x_/AUC_iv_ > 1.2), but CL_R_ following SubQ is 76% after intravenous dosing, which is independent of adsorption issues.

b No statistics are presented, but in a paired comparison could have been significant.

c Average for all oral doses (100, 400, and 800 mg vs 100 mg i.v. bolus)

d The 90% confidence interval values suggest that this finding would have been significant.

e Cilazaprilat measurements. In this study, a highly significant difference was found between the systemic concentration and unchanged drug urinary excretion bioavailability measures.

f Paired data are not provided.

### A Deriving the equations to explain systemic bioavailability measures greater than unity

None of the outcomes listed in Table 2 are possible if measured ${CL}_{oral\;dose\;\left(or\;SubQ\right)}=$ ${CL}_{iv\;bolus}$ is invariably true for linear systems. These measured outcomes result from a very slow absorption process that increases the measured dose-normalized systemic AUC for extravascular dosing compared to intravenous dosing. This is analogous to the sequential metabolism example above in Section V, where slow metabolic clearance of drug to metabolite 1 results in increased apparent MRT of metabolite 2.

#### 1 The present approach

**Fig. 3:**
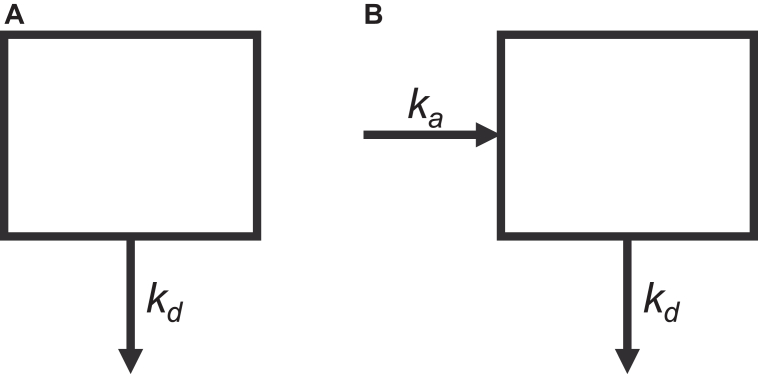
Simple first-order one-compartment body model with metabolic and renal drug elimination for (A) intravenous bolus dosing and (B) oral dosing.

Consider how the field of pharmacokinetics has solved for first-order clearance relationships using differential equations for the past 80 years for a simple oral drug absorption model as depicted in Fig. 3B.

To derive the equation for the amount of drug in the body as a function of time following oral dosing, 2 differential equations for the amount of drug in the body (A_body_) and amount of drug at the absorption site (A_absorp site_) are considered:

$$\frac{{dA}_{body}}{dt}=k_{a}\cdot {F\cdot A}_{absorp\;site}-k_{d}\cdot A_{body\;}\frac{{dA}_{absorp\;site}}{dt}=-k_{a}\cdot A_{absorp\;site}$$

where k_a_ is the first-order absorption rate constant, k_d_ is the first-order elimination rate constant (the sum of the parallel metabolic [k_m_] and renal [k_r_] rate constants), and *F* is the systemic bioavailability. Solving the differential equations for the amount of drug in the body gives:

$A_{body}=\frac{k_{a}\cdot F\cdot Dose}{k_{a}-k_{d}}\cdot (e^{-k_{d}\cdot t}-$ $e^{-k_{a}\cdot t})\text{,}$ which is converted to a concentration equation by dividing by the body volume of distribution, V_body_:

$$C_{body}=\frac{k_{a}\cdot F\cdot Dose}{\left(k_{a}-k_{d}\right)\cdot V_{body}}\cdot \left(e^{-k_{d}\cdot t}-e^{-k_{a}\cdot t}\right)$$

Then integrating the concentration equation over all time to obtain AUC_body,oral, 0→∞_ yields:

(20) $${AUC}_{body,oral,0\to \infty }=\frac{F\cdot Dose}{k_{d}\cdot V_{body}}$$

In contrast for the intravenous bolus dose (Fig. 3A), only 1 differential equation is solved:

$$\frac{{dA}_{body}}{dt}=-k_{d}\cdot A_{body\;}A_{body}=Dose\cdot e^{-k_{d}\cdot t}\;C_{body}=\frac{Dose}{V_{body}}\cdot e^{-k_{d}\cdot t}$$

which when integrated over all time yields

(21) $${AUC}_{body,iv\;bolus,0\to \infty }=\frac{Dose}{k_{d}\cdot V_{body}}$$

Thus, for the traditional approach, the denominator of eq. 21 (the intravenous bolus clearance) is identical to the denominator of eq. 20, which has underpinned the present widely accepted belief that oral dosing does not affect drug clearance. Even if understood that this assumption may not always hold true, the derivation has led to its implicit adoption throughout the field.

A reviewer of this manuscript suggested that the derivation above, using a one-compartment body model, implies that the conclusion is not valid for typical pharmacokinetic data, which often reflect multicompartment kinetics following intravenous bolus dosing. This is not correct. Equation 21 is valid regardless of the number of exponential terms describing CL_iv bolus_. We chose to demonstrate the difference between CL_iv bolus_ versus CL_total_ using a one-compartment body model, because when k_a_ is included in the equation describing drug disposition following an oral dose, frequently the oral concentration data are best described by a 2-exponential equation when the intravenous bolus concentration intravenous data are also described by a 2-exponential equation, and neither of the exponentials in the oral dose equation are k_a_. This is due to the fact, that even using perfect data, computer fits of the data, or any other analysis method cannot differentiate exponential terms unless they differ by at least 2-fold, and with the variability seen with human studies, exponential terms must differ by 3- to 4-fold to be recognized during computer fits of the data. Using a single exponential equation for the intravenous bolus data eliminates this ambiguity, because even if the k_a_ value is almost equal to the disposition rate constant (k_d_ in the demonstration above) both exponentials can be obtained from data described by the oral equation above.

#### 2 The Kirchhoff’s Laws approach

Consider the general Kirchhoff’s Laws eq. 11 for in-series processes:

(11) $$\frac{1}{{CL}_{total}}=\frac{1}{{CL}_{entering}}+\frac{1}{{CL}_{leaving}}$$

For an intravenous bolus dose, ${CL}_{entering}=\infty$, because all drug is assumed to instantaneously enter the systemic circulation, but for an oral dose ${CL}_{entering}={CL}_{gut}$, clearance from the gastrointestinal tract, a parameter not previously considered in pharmacokinetics prior to our introduction (Benet and Sodhi, 2023; Wakuda et al, 2024). For first-order absorption (k_a_) t $\text{hen}\;{CL}_{gut}=k_{a}\cdot V_{gut}$, again introducing a pharmacokinetic volume parameter (volume of distribution in the gastrointestinal tract [*V*_*gut*_]) not previously considered, but which for linear systems is simply the parameter defining the amount of drug in the gut compartment divided by the concentration of drug in the gut compartment. V_gut_ has no more physiologic relevance than the volume of distribution steady state (*V*_*ss*_), but will certainly not be equal to V_ss_, as we previously described (Benet and Sodhi, 2023; Wakuda et al, 2024). Therefore, because CL_leaving_ is the intravenous bolus clearance:

(22) $$\frac{1}{{CL}_{total\;}}=\frac{1}{{CL}_{gut}}+\frac{1}{{CL}_{iv\;bolus}}$$

which when solved gives

(23) $${CL}_{total\;}=\frac{{CL}_{iv\;bolus}\cdot {CL}_{gut}}{{CL}_{gut}+{CL}_{iv\;bolus}}=\frac{{CL}_{iv\;bolus}}{1+\frac{{CL}_{iv\;bolus}}{{CL}_{gut}}}$$

Hence, although we implicitly teach that following oral dosing, absorption has no effect on the clearance measure, this is only true when ${CL}_{gut}\gg {CL}_{iv\;bolus}$. Table III in Wakuda et al (2024) shows that when both systemic and urinary data following both intravenous and oral dosing are available, it is possible to calculate and compare CL_gut_ and CL_iv bolus_. This was done for 9 drugs, with the $\frac{{CL}_{gut}}{{CL}_{iv\;bolus}}$ ratios ranging from a high of 98 for ibuprofen tablets to 1.6 for the cimetidine tablets evaluated by Swedish regulatory scientists (Grahnén et al, 1979). However, the ratio was only low enough for cimetidine, ranitidine, and sodium fluoride for gut clearance to affect the measured total clearance following oral dosing.

#### 3 MRT concepts following oral absorption

Now consider MRT issues for oral absorption with mean absorption time (MAT):

(24) $${MRT}_{oral\;dose}={MRT}_{iv\;bolus}+MAT$$

(25) $$\frac{1}{k_{avg\;oral\;dose}}=\frac{1}{\;k_{avg\;iv\;bolus}}+\frac{1}{k_{a\;avg}}$$

because MRTs are just the inverse of the average (avg) first-order rate constant defining that process (Yamaoka et al, 1979). Note that eq. 25 is the Kirchhoff’s Laws relationship for rate constants following oral absorption; hence, we have been using Kirchhoff’s Laws for more than 40 years, but not realized it. This is the theoretical basis for Kirchhoff’s Laws as applied to pharmacokinetics. Solving eq. 25 gives:

(26) $$k_{avg\;oral\;dose}=\frac{k_{avg\;iv\;bolus}\cdot {\;k}_{a\;avg}}{k_{avg\;iv\;bolus}+k_{a\;avg}}=\frac{k_{avg\;iv\;bolus}}{1+\frac{k_{avg\;iv\;bolus}}{k_{a\;avg}}}$$

Only if k_a avg_ is significantly greater than k_avg iv bolus_ will the terminal concentration-time curve have a slope that is a function of k_avg iv bolus_. Equation 26 mathematically explains the flip-flop model. If calculated, k_a_ is not much greater than the calculated k_iv bolus_; the terminal slope (k_oral dose_) is given by eq. 26.

#### 4 Converting rate constants to clearances

Comparing eq. 26 to eq. 23 allows one to understand why differential equation derivations will provide the correct answer for chemistry but may not for pharmacokinetics. To convert eq. 26 into eq. 23, each rate constant is multiplied by the appropriate volume term, V_gut_ for k_a_ and V_ss_ for k_iv bolus_, demonstrating why differential equation derivations will not yield correct clearance values in pharmacokinetic analyses. However, for chemistry reactions in a beaker, with a fixed volume, differential equation derivations are appropriate, as well as for pharmacokinetic rate constants. However, drug dosing decisions are not based on rate constants; they are based on clearance.

#### 5 Measures of renal clearance support the Kirchhoff’s Laws approach

All pharmacokinetic textbooks state that renal clearance is independent of the route of administration and the value of bioavailability (*F*).

(4a) $${CL}_{R}=\frac{Amount\;excreted\;unchanged}{{AUC}_{driving\;the\;elimination}}$$

Yet in the human crossover studies reported in Table 2 for 1-deamino-8-arginine vasopressin CL_R,SubQ_ was 76% of CL_R,iv_; for cimetidine: CL_R,iv_ = 35.6 ± 12.7 L/h vs CL_R,oral_ = 21.6 ± 10.6 L/h; *P* = .0018 where, as reported in Table 2, CL_R,oral_ was 63% ± 32% of CL_R,iv_, because slow clearance from the absorption site will increase AUC, as predicted by the Kirchhoff’s Laws derivation.

Prior to the application of Kirchhoff’s Laws to pharmacokinetic derivations, the primary explanation for renal clearance being statistically lower for alternate input versus intravenous bolus dosing is that renal clearance was saturated following the alternate input. Yet, for the 1-deamino-8-arginine vasopressin study (Fjellestad-Paulsen et al, 1993), the same dose was given subcutaneously and as an intravenous bolus; hence, saturation cannot be the explanation for the decreased renal clearance. In the cimetidine study (Grahnén et al, 1979), 9 subjects received a 100 mg intravenous dose with mean CL_R,iv_ = 35.6 ± 12.70 L/h, and all 9 received a 400 mg oral dose with a statistically significant decrease in mean CL_R,oral_ = 15.8 ± 4.6 L/h. However, 3 of the 9 also received a 100 mg oral dose with mean CL_R,oral_ = 31.4 ± 7.6 L/h, which was not different than CL_R,iv_ (33.3 ± 3.6 L/h) for these 3 subjects. These 3 subjects plus one additional also received an 800 mg oral dose with mean CL_R,oral_ = 27.2 ± 15.2 L/h vs CL_R,iv_ = 36.6 ± 7.1 L/h for these 4 subjects. Because the 3 subjects receiving the 100 mg oral cimetidine tablet gave an equivalent CL_R,oral_ to the CL_R,iv_ in the same 3 subjects receiving a 100 mg intravenous dose, it could be suggested that the decreased measured clearances at 400 mg and 800 mg were due to saturation. However, another measure can be evaluated. For a drug that is measurably eliminated unchanged in the urine, either systemic or renal elimination saturation will lead to an increased amount of unchanged drug eliminated in the urine as doses increase. For none of the 4 subjects receiving different oral doses was the dose-corrected amount excreted following the highest oral dose greater than the amount excreted for the lowest oral dose. We do not believe that systemic bioavailability for cimetidine exceeding unity is the result of saturable elimination.

In Wakuda et al (2024), we also examined the high systemic bioavailability of 123% for sodium fluoride oral dosing (Ekstrand et al, 1978), but excluded sodium fluoride from Table 2 because the value was not statistically significant (*P* = .055). The lack of statistical significance was due to 1 of the 10 oral dosings in the 6 healthy volunteers giving a renal clearance value that was 80% greater than any of the 12 other measurements in this study, with 2 of the other 12 measurements in this same subject. The sodium fluoride data might also be considered the result of a saturation mechanism as opposed to the increase in area following slow absorption proposed based on Kirchhoff’s Laws. However, none of the 4 subjects receiving multiple oral doses showed any increase in the amount of drug excreted unchanged when dose-corrected measurements from the highest oral dose studied were compared with the lowest dose studied.

## VII What Our published Kirchhoff’s Laws papers explain

1. Why all published experimental data from steady-state IPRL studies fit what has previously been considered as the WSM of hepatic elimination, an unphysiological organ model? None of the published quality experimental data are preferentially consistent with the PTM or DM models of hepatic elimination, though both are regarded as more physiologically relevant than the WSM.
2. Why for linear pharmacokinetic systems, calculated systemic bioavailability can be greater than unity.
3. Why renal clearance can be a function of drug input processes.
4. Why statistically different bioavailability measures can be found for urinary excretion versus systemic concentration measurements in the same study?

Three published papers have questioned the validity of the Kirchhoff’s Laws approach (Korzekwa and Nagar, 2023; Rowland et al, 2023; Siegel, 2023), and were published in response to our first or first and second publications that introduce the application of Kirchhoff’s Laws to pharmacokinetics. After these 3 critiques were published, rather than directly responding we instead further published our explanation for why systemic bioavailability may be greater than unity (Wakuda et al, 2024) and our paper showing the nonsensical outcomes for the mechanistic models of hepatic elimination (WSM, PTM, DMs, and ECM) when determining Kp_uu_ (Benet and Sodhi, 2024a). Subsequently, we very recently responded to these critiques (Benet and Sodhi, 2024b) demonstrating that none of the issues raised were valid, emphasizing that each of the critiques only posed hypothesized theoretical arguments and that none of the critiques actually considered published experimental data. The major objection of Korzekwa and Nagar (2023) was that Kirchhoff’s Laws could not be used to determine the intercompartmental rate constants for multicompartment models, to which Siegel (2023) concurred that Kirchhoff’s Laws could not be used to determine the MRTs for these various hypothesized compartment models. There are 2 good reasons for this. First, the intercompartmental rate constants are never rate-defining processes. Measures of clearance are never equal to intercompartmental rate constants. Second, and more importantly, there is absolutely no relevance of intercompartmental rate constants in determining CL, V_ss_, and MRT from systemic concentration measurements. For all of the different multicompartment models with elimination from different compartments presented by Korzekwa and Nagar (2023) and Siegel (2023), CL, V_ss_, and MRT are identical: $CL=\frac{Dose}{{AUC}_{0\to \infty }};\;{\;V}_{ss}=\frac{Dose\cdot {AUMC}_{0\to \infty }}{{AUC}_{0\to \infty }^{2}}\;and\;MRT=\frac{V_{ss}}{CL}$. Korzekwa and Nagar (2023) and Siegel (2023) are correct. Kirchhoff’s Laws cannot be used to determine intercompartmental rate constants; Kirchhoff’s Laws are only used to derive pharmacokinetic equations that allow useful characteristics of drug disposition to be determined from systemic concentration measurements.

**Fig. 4:**
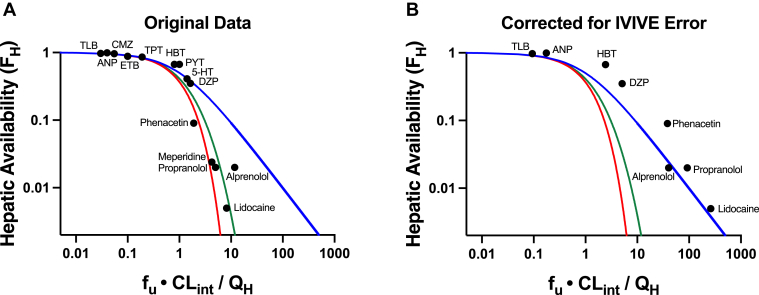
Plots of hepatic availability (*F*_*H*_) vs efficiency number (*f*_*u*_*·CL*_*int*_*/Q*_*H*_) based on (A) originally published analysis, and (B) further corrected for the measured in vitro-in vivo underprediction error. The theoretical clearance relationships are represented with lines in blue (the eq. 15 relationship; previously regarded as the WSM), red (PTM), and green (dispersion model). (A) Data points assuming no error in IVIVE prediction are depicted, based on original analysis from Roberts and Rowland (1986) and Iwatsubo et al (1996). (B) Original data are corrected for the degree of observed in vitro to in vivo (IVIVE) underprediction error, based on human liver microsomal IVIVE data reported by Wood et al (2017). The 5 high extraction ratio compounds included in this analysis (alprenolol, lidocaine, meperidine, phenacetin, and propranolol) are labeled. Additional compounds (low and moderate extraction ratio) are labeled with the following abbreviations: ANP, antipyrine; CMZ, carbamazepine; DZP, diazepam; ETB, ethoxybenzamide; HBT, hexobarbitone; 5-HT, 5-hydroxytryptamine; PYT, phenytoin; TLB, tolbutamide; TPT, thiopental (Adapted from Benet and Sodhi, 2024b,c).

Of particular relevance to point (1) above, a major topic of criticism by Rowland et al (2023), is Fig. 4A, which was presented by both Professors Rowland and Sugiyama as supporting the PTM and DM mechanisms of hepatic elimination at the September 2023 International Society for the Study of Xenobiotics symposium, “50 Years of Clearance Prediction.” Both speakers cited studies from their laboratories in presenting versions of Fig. 4A (Roberts and Rowland, 1986; Iwatsubo et al, 1996), where the *y*-axis values are published hepatic availability *F*_*H*_ measures, experimentally determined from ex vivo IPRL studies. However, the *x*-axis values are calculated efficiency numbers (*f*_*u*_ ⋅ *CL*_*int*_/*Q*_*H*_), which were determined by combining the experimentally utilized *Q*_*H*_ and *f*_*u*_ values from the IPRL study with a predicted in vivo *CL*_*int*_ that is based on in vitro-in vivo extrapolation (IVIVE) of in vitro *CL*_*int*_ measures from a different study. As we recently noted (Benet and Sodhi, 2024b), “Notably, the calculated in vivo *CL*_*int*_ values assume that IVIVE has no error, and that the in vitro *CL*_*int*_ value may accurately predict the in vivo *CL*_*int*_. In the last century, it may have been believed that IVIVE would give quantitatively accurate values, but we know today from multiple studies that this is not true and that throughout the field, as presently employed, IVIVE consistently underpredicts the in vivo measured experimental clearance values (Sodhi and Benet, 2021). At the time that Fig. 4A was originally presented the authors understandably may not have appreciated this difference. But subsequently, both speakers have published with their colleagues that they recognize that the previous assumption of the accuracy of IVIVE is incorrect” (Chiba et al, 2009; Rowland and Pang, 2018).

In Fig. 4B, reproduced from Benet and Sodhi (2024b,c), we replotted the x-values for all of the data points where IVIVE error data were available using the degree of IVIVE underprediction for human microsome experiments, as reported by Wood et al (2017). It is instructive that when the IVIVE underprediction is accounted for, all of the data appear to be best described by eq. 15 (blue line), previously regarded as the WSM. Thus, we maintain that although the field believes that the WSM is unphysiological and that the PTM and DMs are more representative of liver elimination for high-clearance drugs, there are no quality steady-state perfused liver experimental studies available demonstrating that data are best described by the PTM and DMs when only systemic concentrations are measured. Equation 15 continues to best describe all valid experimental data available in the published literature independent of any mechanistic model of hepatic elimination.

## VIII Ongoing discoveries and further applications of Kirchhoff’s Laws

Above we have detailed our published peer-reviewed papers and their application in explaining previous published experimental data as summarized in section VII. However, as we note in the Introduction, our discoveries and applications of Kirchhoff’s Laws are ongoing. Here, we briefly describe work under review, presented in submitted meeting abstracts, and further topics we plan to address. Most of this material has not yet been subject to peer review, but we believe that readers may find a preview of these applications to be useful.

### A Increased response effects for slow-release pharmaceutical formulations

If slow entering clearance from the delivery site for a dosage formulation can increase the dose-corrected AUC compared to an intravenous bolus dose, as demonstrated above, this could also be true in comparing dose-corrected AUC for ER oral dosage forms versus IR oral dosage forms. Because for the great majority of drugs an increase in AUC can result in an increase in the pharmacodynamic effect, it is now possible to explain multiple published studies that demonstrate increased pharmacodynamic response for equivalent dose ER vs IR formulations. We recently presented this analysis in a published abstract (Tiitto et al, 2024) and will fully describe it in detail in a future publication. However, here we cite Lukacsko et al (2004) and reproduce Table 2 from that paper, here as Table 3.

**Table 3:** Inferential analysis results for percent change in LDL-cholesterol, HDL-cholesterol, total cholesterol, and triglycerides from baseline to endpoint in a population treated with 20 mg lovastatin (adapted from Lukacsko et al, 2004)

Treatment 20 mg (n = 149)	Percent Change – Least Square Mean ± Standard Error (95% Confidence Interval)			
	LDL Cholesterol	HDL Cholesterol	Total Cholesterol	Triglycerides
ER lovastatin	–26.4 ± 1.06 (–28.5, –24.3)	4.1 ± 1.04 (2.0, 6.1)	–19.1 ± 0.83 (–20.7, –17.4)	–7.4 ± 2.14 (–11.7, –3.2)
IR lovastatin	–23.1 ± 1.06 (–25.2, –21.0)	4.3 ± 1.04 (2.3, 6.4)	–17.2 ± 0.83 (–18.8, –15.5)	–10.4 ± 2.14 (–14.7, –6.2)
ER vs IR	–3.3 ± 1.08 *P* = .0028	–0.2 ± 1.28 *P* = .8584	–1.9 ± 0.91 *P* = .0355	3.0 ± 2.81 *P* = .2867

HDL, high-density lipoprotein; LDL, low-density lipoprotein.

All numbers are from a two-way ANOVA for the percent change with treatment and center as factors.

As seen in Table 3, the authors reported a significant decrease in low-density lipoprotein-cholesterol (*P* < .0028) and total cholesterol (*P* < .0355) for a 20 mg lovastatin ER formulation vs a 20 mg IR formulation, with no significant changes in high-density lipoprotein cholesterol and triglycerides. In a separate study with a different population (Davidson et al, 2002), an increase in lovastatin AUC_0→24_ was observed for the ER vs the IR formulation, on day 1 and day 28, leading to the pharmacodynamic increase for the ER formulation in Lukacsko et al (2004). Our to-be-submitted manuscript will include many other examples.

### B The potential of developing high hepatic clearance drugs via controlled release

When a new molecular entity is predicted to exhibit high clearance in humans, pharmaceutical sponsors almost universally search for similar-acting backup compounds that will demonstrate low clearance. In a manuscript that was published (Benet et al, 2024) after the initial submission of this review, we show that there can be marked advantages to developing controlled release formulations of high-clearance drugs for nonoral routes of administration. These advantages include not being concerned with the following: (1) saturable nonlinear kinetics, (2) significant pharmacogenomic differences, (3) drug-drug induction mechanisms, and (4) in many cases drug-drug inhibition interactions. This is due to the ability of a drug sponsor to design clearance based on slow drug release, independent of the pharmacokinetic characteristics for high-clearance compounds, where clearance from the dosage form becomes the drug clearance from the patient. Recognition of this principle results directly from our application of Kirchhoff’s Laws to derive rate-defining clearance and rate constant elimination processes independent of traditional differential equation derivations.

### C The application of Kirchhoff’s Laws to saturable, Michaelis-Menten, pharmacokinetics

All of our published papers only apply to linear kinetic systems. However, in an accepted abstract presentation for the September 2024 ISSX/JSSX Meeting in Hawaii, USA (Benet and Sodhi, 2024d), we demonstrate the derivation of the Michaelis-Menten equation for in vivo saturable metabolic processes. Following the Kirchhoff’s Laws derivation for in-series clearance steps, we derive the inverse of the in vivo saturable hepatic clearance equation by adding the inverse of the 3 rate-defining processes.

(27) $$\frac{1}{{CL}_{H,saturable}}=\frac{1}{\frac{V_{\max}}{K_{M}}}+\frac{1}{\frac{V_{\max}}{\left[S\right]}}+\frac{1}{Q_{H}}$$

where [S] is the substrate concentration with units of mass per volume, V_max_ has the units of mass/time and is the maximum rate of substrate elimination when metabolism is saturated, and K_M_ has the units of mass per volume and is the concentration of substrate at the half-maximal rate of elimination. Based on eq. 27, one can recognize that there may be in vivo saturable metabolic processes where hepatic blood flow is considered, which we show in our ISSX/JSSX abstract (Benet and Sodhi, 2024d) is relevant for ethanol. Most importantly, we recognized that it is not possible to incorporate hepatic blood flow into a Michaelis-Menten equation using differential equations. In addition, due to the potential differences in the in vivo volumes of distribution for the substrate, enzyme, and enzyme-substrate, we suspect that frequent measurements of enzyme-substrate on and off rates in vitro may not be predictive in vivo. This may also be relevant to predicting the extent of in vivo drug-drug interactions based on in vitro measures both for saturable and first-order processes. These concepts are further explained in a recently submitted manuscript on the topic of nonlinear kinetics and Kirchhoff’s Laws.

### D Explaining the decrease in clearance for high hepatic extraction ratio drugs as a function of increasing zero-order infusion times

Just as increasing MAT may affect the rate constant for elimination (eq. 24) and the elimination clearance, prolonged infusion times resulting in increased mean input times can affect the rate constant for elimination. This will be most obvious for high hepatic clearance drugs such as lidocaine. A number of in vivo studies in humans and animals have shown marked decreases in lidocaine clearance with prolonged zero-order infusions as may be best represented by the study of Ochs et al (1980) in 11 healthy subjects. Three of these subjects were crossed over and clearance of lidocaine was determined following a single 4 minute infusion (24.3 ± 6.1 mL/min/kg) and following a 36-hour zero-order infusion of lidocaine (10.5 ± 1.0 mL/min/kg). Even though there are crossover data for only 3 subjects, the paired decrease in clearance is significant (*P* = .046). Present pharmacokinetic theory cannot explain these results, but in a to-be-published paper, we will show how Kirchhoff’s Laws can explain the outcome and predict a potential change in clearance with increasing zero-order infusion time based on MRT principles.

## IX Conclusions and final remarks

There is no doubt that the adapted Kirchhoff’s Laws approach to deriving clearance and overall rate constant equations as presented in this review has been viewed as very controversial, with the initial reaction of many knowledgeable investigators being to question its applicability. However, as presented here, this new methodology can explain numerous examples of in vivo data that were viewed in the past as either fraught with experimental errors, often ignored, or in many cases not published as there was no logical explanation for the findings. Like our entire field throughout its entire existence, we too could not explain these supposedly anomalous data until recently. As we describe here, the error in present pharmacokinetic theory is in converting differential equation derivations for chemistry rates of reaction (in terms of rate constants and amounts) to define clearance and concentrations by dividing by a single volume of distribution for all in vivo processes. This approach works in chemistry because all reactions occur in a fixed volume of fluid containing all the reactants, and for defining rate constants in pharmacokinetics, because the volume of distribution is unimportant. The most important outcome from these analyses is the recognition that slow input from a site of administration can increase the measured AUC, contrary to the belief held for the past 80 years that input has no effect on measured systemic concentrations. We emphasize that the field cannot be taken to task for this error, because until our publications there was no methodology for deriving clearance equations except via differential equations. What led to the discovery of the Kirchhoff’s Laws approach? The origin was our recognition that in the WSM derivation, it did not seem proper that the hepatic clearance determination in systemic fluids could be influenced by hepatic blood flow while having the clearance in the liver not influenced directly by hepatic blood flow (Benet et al, 2018b). Of note, the senior author of this review was initially involved in the WSM derivation (Rowland et al, 1973), highlighting that it took almost 50 years to recognize this differentiation. However, few of the leaders in our field agreed with this point. The big advance for us came when we reviewed all of the published experimental steady-state IPRL data (Sodhi et al, 2020b) and recognized that none of the published quality studies preferentially supported the PTM and DM models. This finding initially received very little attention, because other influential investigators (Pang et al, 2019) agreed with our analysis (Benet and Sodhi, 2020, 2022; Benet et al, 2021; Sodhi and Benet, 2021), that the experimental data did surprisingly appear to all be consistent with the WSM, a mechanistic model that is universally agreed not to be physiologically relevant. However, in 2022, we discovered that we could derive the equation believed to be the WSM (eq. 15) by adapting Kirchhoff’s Laws from physics, completely independent of any mechanistic model of hepatic elimination (Pachter et al, 2022) and independent of the need for a differential equation derivation. Thus, we introduced a previously unrecognized methodology for deriving clearance equations. Further, we had an explanation for why all published IPRL data appeared to follow a single universal clearance relationship.

Before we published further papers justifying our methodology, 3 publications questioned the validity of our adapted Kirchhoff’s Laws approach, as we reviewed above. After that, further publications were initially increasingly difficult to publish, as reviewers for each of our subsequent papers continued to recommend rejection, even when our responses were submitted to the same journal as the critiques, because our concepts did not agree with pharmacokinetic theory nor with the opinions of highly regarded colleagues in the field, on topics that have been accepted by our field since its founding. We recognized that what we were proposing would be difficult for many investigators, reviewers, and especially for editors, who had to follow the majority recommendations of their reviewers. The strategy we employed after rejection from an initial journal when submitting to a second journal, was to submit the final rejection reviews, sometimes after 2 revisions, along with our rebuttal response explaining why these reviews were not valid, accompanying the further revised manuscript to a second journal.

A further big breakthrough with respect to the lack of utility of the mechanistic models of hepatic elimination when only measuring systemic drug concentrations occurred most recently (Benet and Sodhi, 2024a) when we carried out an analysis that had not been previously considered. We critically examined the general equation for the derivation of all of the mechanistic models of hepatic elimination (eq. 18a) and showed that when determining Kp_uu_ for each of the mechanistic models, including the ECM, the value of Kp_uu_ cannot exceed 1 and that for each of the models it is a function of F_H_, the model-independent measure of hepatic bioavailability. It may take some time for this analysis to be fully appreciated because mechanistic models of hepatic elimination have been entrenched in our field and utilized in physiologic-based pharmacokinetic models for the past 50 years. However, we believe pharmacologists and pharmaceutical scientists will recognize that a relationship between Kp_uu_ and F_H_ is not valid and the idea that hepatic Kp_uu_, can never exceed unity is not consistent with the physicochemical properties of most drugs.

In summary, eq. 15 is not the WSM, which our field has inadvertently been led to believe was correctly adapted from Chemical Engineering but was in fact based on errors in traditional pharmacokinetic theory. Chemical engineers do not believe that the rate of reaction within a reactor is independent of the flow rate of the reactants into the reactor. When only systemic concentrations are measured, eqs. 15 and 16 are the equations describing hepatic clearance independent of any mechanistic model.

Following our initial Kirchhoff’s Laws publication (Pachter et al, 2022), we recognized that the in-series relationship utilized to determine hepatic clearance, eq. 11, encompassing clearance entering and clearance leaving, could also be applied to nonintravenous bolus pharmacokinetic equations of clearance (Benet and Sodhi, 2023; Wakuda et al, 2024) as given in eq. 23. This allowed us to explain a number of clinical in vivo findings that had been considered to result from poor experimental procedures. These include the following possibility: (1) that bioavailability measured using systemic concentrations can significantly exceed unity for slow input delivery of drug; (2) that renal clearance for such slow input formulations can be significantly less than that seen for a comparable intravenous bolus dose without having to assume that the renal clearance of the alternate input dose undergoes saturation in the renal tubule; (3) that bioavailability calculated using urinary excretion of unchanged drug in the urine can be significantly less than the bioavailability in the same study calculated using systemic concentrations; (4) that one can explain and predict why pharmacodynamic effects following administration of an ER dosage form, including oral dosing, can be significantly greater than that seen for administration of a comparable IR dosage form.

As we detail in section VIII of this review, the application of our adapted Kirchhoff’s Laws approach is ongoing. We briefly introduced in section VIII further applications that are the subject of under review or to-be-published applications for deriving nonlinear clearance equations, why there may be a benefit to developing nonoral controlled release formulations of high hepatic clearance drugs and an explanation for the many papers reporting in vivo lidocaine clearance significantly decreasing with increasing zero-order infusion times. We expect that there will be many further applications of Kirchhoff’s Laws that we and others will discover. Obviously, a significant future benefit will be seen in the teaching of pharmacokinetics because clearance and total rate constant coefficients of proportionality may be derived independent of differential equations, by just recognizing parallel and in-series rate-defining processes.

## Conflict of interest

The authors declare no conflicts of interest.
